# Use of Cryotherapy for Managing Epistaxis in the First Aid Setting: A Scoping Review

**DOI:** 10.7759/cureus.14832

**Published:** 2021-05-04

**Authors:** David Berry, Jestin N Carlson, Eunice Singletary, David A Zideman, Jennifer Ring

**Affiliations:** 1 Kinesiology, Saginaw Valley State University, Saginaw, USA; 2 Emergency Department, Saint Vincent Hospital, Erie, USA; 3 Emergency Medicine, University of Virginia, Charlottesville, USA; 4 Pre-Hospital Emergency Medicine, Thames Valley Air Ambulance, Oxford, GBR; 5 Australian Resuscitation Council, Australian and New Zealand Committee on Resuscitation (ANZCOR) Evidence Reviewer, East Melbourne, AUS

**Keywords:** epistaxis, nasal bleeding, cryotherapy, ice packs, cold packs, first aid, prehospital, lay provider

## Abstract

Epistaxis, or nosebleed, is bleeding from the nostril(s), nasal cavity, or nasopharynx. Anterior nasal bleeding is the most common location for spontaneous nontraumatic epistaxis and is commonly treated with manual compression to the nasal alae. Cryotherapy is also routinely recommended in conjunction with manual compression in the first aid and ED setting.

We performed a scoping review on behalf of the International Liaison Committee on Resuscitation First Aid Task Force guided by the Preferred Reporting Items for Systematic Reviews and Meta-Analyses extension for scoping reviews (PRISMA-ScR). We searched Embase, Cochrane, and PubMed databases for published studies, without date restrictions, and we searched the gray literature using Google.com and Google Scholar. The websites of selected resuscitation councils were searched for guidelines relating to the management of epistaxis. References from included studies were hand-searched. Our published and gray literature search identified 1255 and 61,315 records, respectively. After removing duplicates and following selection criteria, we included 21 records from the published literature and 11 records from the gray literature.

Our scoping review found that most of the published studies and website documents focused on managing nontraumatic epistaxis in the first aid setting. They provide recommendations for the use of cryotherapy based on expert opinion or indirect evidence extrapolated from cryotherapy-associated changes in nasal submucosal temperature, nasal blood flow, and nasal blood volume in healthy subjects (three studies). We did not identify any prospective, randomized trials comparing the efficacy of cryotherapy as an intervention for nontraumatic epistaxis in the first aid setting. The limited literature identified in this scoping review does not support the development of a systematic review but highlights the need for future research to better understand the role of cryotherapy in the first aid setting.

## Introduction and background

Epistaxis, nasal or nosebleed, is bleeding from the nostril(s), nasal cavity, or nasopharynx caused by the rupture of a blood vessel within the nasal mucosa [1,2]. Rupture of the blood vessels can be spontaneous, initiated by trauma, secondary to certain medications, and/or secondary to other comorbidities or malignancies [3]. Approximately 60% of the population worldwide will experience spontaneous epistaxis, but only 6-10% of those affected will need medical care [4,5]. However, the true prevalence of epistaxis is challenging to measure because most individuals with nasal bleeding experience a minor bleeding episode and opt for conservative self-treatment (first aid) at home [5,6], as these incidents are rarely reported [7]. Spontaneous nosebleeds are particularly common in children between two years and 10 years of age and are likely due to digital nasal trauma or mucosal irritation.

In the first aid setting, direct pressure (compression) to the lower third of the nose (Kiesselbach's area or Little’s area) is recommended as first-line care for patients with active nasal bleeding [5,8-10]. Cryotherapy (i.e., ice bags, ice packs, ice cubes, ice collars, commercial ice, frozen vegetable packs, ice collars) is routinely recommended in the gray literature in conjunction together with other therapies (i.e., compression) as a means of supportive or conservative care [9,11-17]. Investigators from the 1930s proposed that cooling localized areas of skin, including the hands, feet, or back resulted in shrinkage of the nasal mucosa, likely due to reactive vasoconstriction [18]. This may, in turn, decrease mucosal blood flow and aid in hemostasis during a nosebleed. However, the usefulness of cryotherapy applied alone or in conjunction with compression to control active bleeding for epistaxis is unclear.

The 2000 American Heart Association Emergency Cardiac Care (ECC) guidelines for first aid directs first aid providers to manage epistaxis with nasal compression; cryotherapy was not mentioned as a first aid care strategy and no further reviews or first aid treatment recommendations have since been published by the International Liaison Committee on Resuscitation (ILCOR) [19]. Therefore, we performed a scoping review to identify the current state of the literature regarding cryotherapy as an adjunctive or individual intervention for the management of spontaneous nontraumatic epistaxis in the first aid setting by non-medical providers (first aid providers, lay responders) and to answer the population, interventions, comparators, outcomes, study design, timeframe (PICOST) question: among adults and children receiving first aid for spontaneous nontraumatic epistaxis, does cryotherapy alone or combined with nose pinching, compared with nose pinching alone, change outcomes of hemostasis, time to hemostasis, reduction of nasal blood volume, reduction of pain, need for follow-up care, adverse events, recovery time, and reduction of intranasal swelling?

## Review

This scoping review was performed as part of the ILCOR continuous evidence evaluation process, conducted by the ILCOR First Aid Task Force Scoping Review team for the 2021 Consensus on Science with Treatment Recommendations (CoSTR).

Scoping search strategy

We performed four structured searches: (1) published literature using Embase, Medline, and Cochrane; (2) clinical practice guidelines and position statements searches from professional organizations using PubMed.gov; (3) gray literature search using Google.com, Google Scholar, and the constituent resuscitation council (and sub-councils) websites aligned with ILCOR; and (4) hand searching of secondary resources identified from reviewed manuscripts on nontraumatic epistaxis was conducted as required.

Published literature

We created a search strategy in consultation with a senior health researcher (DCB, EMS, JR) for the published literature. The initial search strategy (Embase, Medline, and Cochrane) included all years and all languages as long as there was an English abstract; unpublished studies (e.g., conference abstracts, trial protocols) were excluded unless subsequently picked up in the gray literature search (Appendices 1-8). The initial published literature search was conducted on July 13, 2020, and updated on January 14, 2021 (Appendices 1-4).

We conducted a clinical practice guideline and position statement search using PubMed.gov on December 19, 2020, to specifically identify clinical practice guidelines and position statements from professional organizations (Appendix 5). The search was inclusive for all years and all languages as long as an English abstract was available.

Gray literature

We conducted a gray literature search of Google.com on December 21, 2020 (DCB, JNC), to identify clinical practice guidelines related to first aid for spontaneous nontraumatic epistaxis (Appendix 6). The search was inclusive for all years and limited to the first 50 websites. A second gray literature search of Google Scholar was conducted (DCB, JNC) on December 28, 2020, to identify relevant literature (Appendix 7). The search was inclusive for all years and limited to the first 100 sites per search string. We conducted a final gray literature search on December 28, 2020 (DCB, JNC) to examine the eight-constituent resuscitation council (and sub-councils) websites aligned with ILCOR (Appendix 8). We hand searched the organizations' websites for position or guideline statements addressing the first aid management of epistaxis. We manually reviewed references from all included studies.

Clinical question

Our population included adults and children receiving first aid for spontaneous nontraumatic epistaxis appropriate to the first aid setting (Table 1). We included all human studies where cryotherapy or cryotherapy with nose pinching (manual or mechanical) was examined. Randomized controlled trials (RCTs) and non-randomized studies (non-randomized controlled trials, interrupted time series, controlled before-and-after studies, cohort studies) were eligible for inclusion. Case series and gray literature were also eligible for inclusion. Critical outcomes for the review included hemostasis (yes/no), time to hemostasis (minutes), and reduction of nasal blood volume or area (mL or cm^2^ or cm^3^). Important outcomes included reduction of pain (yes/no), need for follow-up care (yes/no), adverse events (yes/no), recovery time (days/min), and reduction of intranasal swelling or area (mL or cm^2^ or cm^3^). Given the nature of this scoping review, we also considered outcomes as evaluated in newly included articles.

**Table 1:** Scoping review inclusion and exclusion criteria.

	Inclusion criteria	Exclusion criteria
Population	Adults and children, nontraumatic spontaneous, anterior epistaxis	Infants (< 1-year-old) nontraumatic, idiopathic epistaxis, posterior epistaxis, chronic epistaxis
Intervention	Cryotherapy or cryotherapy with nose pinching (manual or mechanical)	Any intervention not feasible in a first aid setting (e.g., commercial ice packs or machines), intranasal cryotherapy application
Comparison	Nose pinching alone	Any intervention not feasible in a first aid setting (e.g., cautery, intranasal, packing, silver, tranexamic acid)
Outcome	Critical outcomes; time to hemorrhage control (minutes), hemostasis (yes/no), critical, reduction of nasal blood volume (mL, cm^2^, cm^3^), reduction of pain measured via pain scale (pain scale). Important outcomes; the need for follow-up care (yes/no), adverse events (yes/no). Less important outcomes; recovery time (days/min), reduction of swelling (volume).	
Study design	Randomized controlled trials (RCTs) and non-randomized studies (non-randomized controlled trials, interrupted time series, controlled before-and-after studies, cohort studies)	Unpublished studies (e.g., conference abstracts, trial protocols) were excluded unless subsequently picked up in the gray literature search
Timeframe and language	All years. All languages, as long as an English abstract is available.	Articles in a language other than English, for which no English abstract is available or unable to translate.
Setting	First aid provider in the first aid setting	Healthcare providers in emergency departments or clinics.

Screening

For the published literature search, two independent reviewers (DCB, JNC) screened the title and abstract of each article. Two independent reviewers (DCB, JNC) then performed a full-text review of potential articles to determine the final articles to be included. One reviewer (DCB) performed the initial search and identified potential sources from the gray literature search. Two reviewers (DCB, JNC) then reviewed these sources to identify any additional key sources of information. In cases of inconsistency, an additional reviewer (EMS) adjudicated the discrepancy. We present descriptive summaries of the final included manuscripts and gray literature.

Results

**Figure 1:**
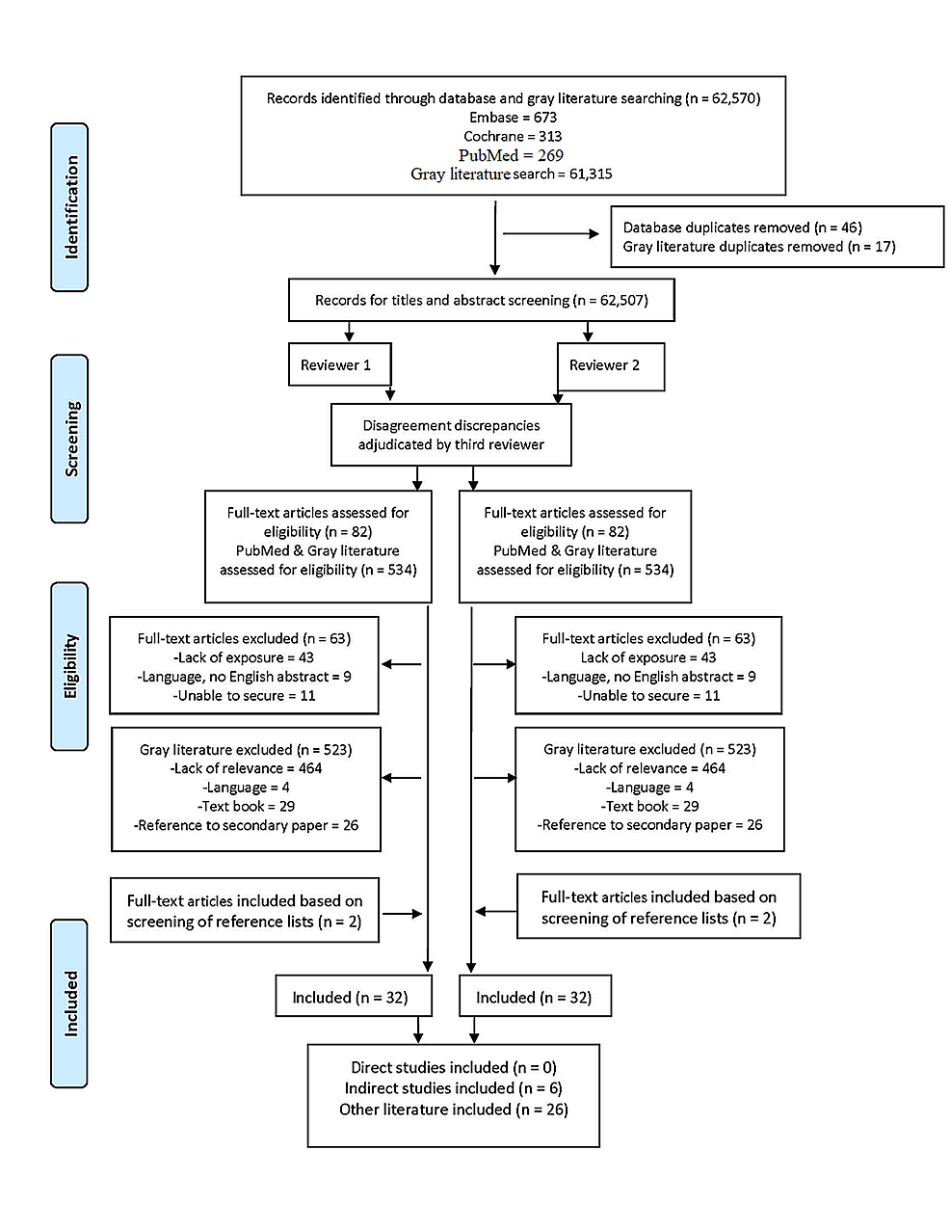
PRISMA diagram of included studies. PRISMA: Preferred Reporting Items for Systematic Reviews and Meta-Analyses

Our published literature search identified 1255 records. After removing duplicates (n=46) and title and abstract screening by two reviewers (DCB, JNC), we identified 82 articles for further review. No studies directly addressed the clinical question; however, within the boundaries of a scoping review, we identified 19 records for inclusion (Figure 1), six of which were considered indirect evidence relating to the clinical question (Table 2). The broad gray literature search identified 61,315 potential additional sources of information. The search was limited to 551 records (Figure 1), and after removing duplicates (n=17), we identified 534 records. 11 records were selected for inclusion in this scoping review.

**Table 2:** Indirect evidence study characteristics and findings. L, left; R, right

Author, year, country	Study design	Population	Intervention	Control	Outcome	Findings on our outcomes, as presented in the article
Ozturk et al., 2014, Turkey [20]	Observational	15 patients (mean age=28.8; 9 female) at least 18 years of age with no nasal symptoms within 3 weeks, and not pregnant.	Nasal dorsal skin cooling using two ice packs applied to left (L) and right (R) side of nose for 10 minutes (n=15).	No application, baseline	Cross-sectional area (cm^2^) and nasal cavity volume (cm^3^) via acoustic rhinometry	Mean values for the sum of the L and R first minimal cross-sectional area and second minimal cross-sectional area revealed no statistical differences, for either parameter at any between any intervals. Means values for nasal cavity volume revealed no statistical differences, for any parameter, between any intervals.
Porter et al., 1991, United Kingdom [21]	Cross-over, Randomized	16 healthy subjects (mean age=32, range 25-40) with no history of the nasal disease, previous nasal surgery or symptoms, and a normal rheoscopic examination.	Ice contained with a surgical glove applied to forehead or mouth for 3 minutes each (n=16).	Same, but at body temperature for 3 minutes each.	Nasal mucosal blood flow, measured in flux (velocity and concentration of the moving blood cells)	Oral ice packs produced a significant decrease in nasal mucosal blood flow (p<0.05, average decrease=23% {standard error=5.9}) compared to control (average decrease=5%; standard error not calculated/provided). Oral ice packs produced a fall in flux in 9 of 16 (56%) subjects, a rise in 1 (6%), and 6 (37%) experienced no change. Ice packs to the forehead produced a fall in flux in 1 of 16 (6%) subjects, a rise in 1 (6%).
Porter, 1991, United Kingdom [22]	Cross-over, Randomized	13 healthy subjects (mean age=30, range 25-40) with no nasal disease or treatment.	(a) Ice pack wrapped in paper toweling held to the forehead by subject for 15 minutes. (b) Ice cubes sucked into the mouth for 15 minutes. (c) Combination of (a) and (b) for 15 minutes.	No application, baseline	Nasal submucosal temperature (°C)	A significant difference between the nasal submucosal temperature ice pack to the forehead (a) compared to ice cubs in the mouth (b) (p=.0.026), favoring ice cubes alone. A significant difference between nasal submucosal temperature in the ice pack to the forehead compared to the combined stimulus (c) (p=.0.006), favoring combined stimulus. In all subjects (n=13, 100%) ice cubes in the mouth (b) produced a lower nasal submucosal temperature. The ice pack to the forehead (a) produced a decrease in nasal mucosal temperature in 7 of 13 (53%) subjects.
Scheibe et al., 2006, Germany [23]	Cross-over	15 healthy subjects (range 25-40, 7 female) with no reported breathing difficulties, acute nasal allergies, or acute rhinitis; nasal endoscopy by an ENT specialist revealed no pathology.	Ice collar (4°C) placed onto neck region for 10 minutes.	No application, baseline	Nasal blood volume via optical rhinometry (measured in nm) for the whole nose and at the septum, randomized.	A significant (p<0.01) decrease in nasal blood volume for regional measurements at the septum. Decrease in nasal blood volume at nasal septum was, on average, observed after approximately 2 minutes t_1_=111 sec ± 73 sec); decrease reached its maximum after approximately 6 minutes (t_2=_337 sec ± 119 sec).
Teymoortash et al., 2003, Germany [24]	Cross-over	56 healthy subjects (mean age=30, range 17-48) with normal rhinoscopy and no history of nasal allergy or acute or recurrent symptoms of rhinitis.	Ice pack applied all-round the neck for 5 minutes.	No application, baseline	Nasal mucosal microcirculatory blood flow via laser Doppler flowmetry, nasal mucosal blood content (indirectly via conventional computer-aided anterior rhinomanometry by measuring alternations in nasal airflow and airway patency).	Following cold application, nasal mucosal blood flow decreased from 1368.8 ± 927.9 to 1130.5 ± 792.2), (p=0.11). Total nasal inspiratory airflow before the application was 513.9±190.4 cm^3^/s, after exposure to cold 471.5±164.6 cm^3^/s (p=0.08). Total nasal expiratory airflow before the application was 474.2±211.7 cm^3^/s, after exposure to cold 443.1±162.4 cm^3^/s (p=0.30).
Yamagiwa et al., 1990, Denmark [25]	Cross-over	10 healthy subjects (mean age=21±11.0, range 24-54) with no significant complaints or rhinoscopically overt nasal abnormalities.	(a) Feet cooling (both) in large tub (0-4°C) immersed 30 cm from heel for 5 minutes, (n=10). (b) One hand and forearm cooling in a bucket (0-4°C) immersed to around 23 cm from the middle fingertip for 5 minutes (n=9).	No application, baseline	Nasal cavity volume (mL) rhinometry for L and R cavities.	Foot cooling arm. In the exposure period, nasal airway volume was significantly higher than preexposure values in 4 of 10 (40%) subjects, none showed significantly lower values. Hand cooling arm. In the exposure period, nasal airway volume was significantly higher than preexposure values in 1 of 9 (11%) of subjects, lower in 2 of 9 (22%), and no difference in 6 of 9 (66%)

The search strategy yielded many scientific publications and information records in the gray literature; however, after subsequent review, none of these records directly addressed our PICOST. Therefore, there were insufficient studies identified to support a more specific systematic review. As a scoping review, we sought to include indirect evidence and gray literature to identify knowledge gaps and scope the body of literature relative to epistaxis management with cryotherapy in the first aid setting.

Indirect evidence of cryotherapy to the nasal area

Six indirect experimental studies were identified from the published literature search [20-25]. These examined the effects of cryotherapy on nasal mucosal blood flow [21,22,24], nasal submucosal temperature [22], nasal blood volume [23], nose and nasal congestion and nasal cavity volume [20], nasal airflow and patency [24], and nasal airway volume [25]. Table 2 provides an overview of study characteristics and data findings, all in healthy subjects.

Two randomized (i.e., intervention counterbalanced) cross-over studies enrolling 29 adults evaluated the effect of ice applied to the forehead, or in the mouth, or a combination of both, on nasal mucosal blood flow using a laser Doppler flowmeter (measured in flux) [21] and measured nasal submucosal temperature (°C) using a thermocouple inserted into the submucosa of the inferior turbinate [22].

The first study (n=16 adults) assessed nasal mucosal blood flow following the application of a surgical glove filled with ice applied to the forehead for three minutes compared with the placing of a surgical glove filled with ice into the mouth for three minutes [21]. The application of the ice packs within the mouth was reported to produce a significant decrease in nasal mucosal blood flow (p<0.05, average fall 23% {standard error 5.9}) compared with the control (average decrease of 5% standard, error not calculated). Oral ice packs produced a decrease in flux (velocity and concentration of the moving blood cells) in nine out of 16 (56%) subjects, one (6%) experienced an increase in flux, and in six (37%), there was no change.

The second study (n=13 adults) assessed nasal submucosal temperature at the inferior turbinate following 15-minute periods of cryotherapy using ice packs (ice packs alone) wrapped in a paper towel applied to the forehead, ice cubes (ice cubes alone) “sucked” in the mouth, and a combination of both therapies (ice pack + ice cubes) [22]. A significant difference in nasal submucosal temperature was reported between the "ice pack alone" and the "ice cubes alone" groups (p=0.026), favoring a lower nasal submucosal temperature in the "ice cubes alone" group (data not provided). There was a significant submucosal temperature difference between the "ice pack alone" group and the combination group (p=0.006), favoring a lower nasal submucosal temperature in the combination group (data not provided). A decrease in submucosal temperature was demonstrated in 100% (13/13) of participants in the ice cubes alone group compared with seven out of 13 (54%) participants in the ice pack alone group.

Teymoortash et al. performed an observational before and after study with 56 healthy adults comparing no use of ice packs (before) with ice packs (no specific description provided) applied for five minutes all around the neck (after) [24]. Outcomes included nasal mucosal microcirculatory blood flow in Kiesselbach’s area measured via laser Doppler flowmeter and nasal mucosal blood content estimates using a computer-aided anterior rhinomanometry, made by alterations in nasal airflow and airway patency. The fall in nasal mucosal microcirculatory blood flow before (1368.8 ± 927.9 arbitrary units) and after (1130.5 ± 792.2) ice application was not statistically significant (p=0.11; mean difference {MD} -238.3, 95% confidence interval {CI} -559.6 to 83.0). The fall in total nasal inspiratory airflow before (474.2 ± 211.7 cm^3^/s) and after (443.1 ± 162.4 cm^3^/s) ice application was also not statistically significant (p=0.30, MD -31.1, 95% CI -101.7 to 39.5).

Scheibe et al. undertook a cross-over before and after study with 15 healthy adult participants comparing no use of an ice collar (pre) with the application of an ice collar (4°C) to the neck (post) for 10 minutes, on nasal blood volume for the whole nose and septum (Kiesselbach’s area) using optical rhinometry [23]. The authors reported no significant effect in whole nose blood volume using ice collars to the neck. A significant (p<0.01) decrease in nasal blood volume was reported for regional measurements at the septum. However, data were only reported graphically, and we were unable to extract exact values. The reduction in nasal blood volume at the nasal septum was, on average, observed after approximately 120 seconds (111 ± 73) and reached its maximum after about 360 seconds (337 ± 119).

Ozturk et al. undertook an observational study enrolling 15 healthy adults comparing no application of a cold compress (before) with the application of a cold compress to the left (L) and right (R) nasal dorsal skin for five minutes and 10 minutes, on the outcomes of the nose and nasal congestion (cross-sectional area, cm^2^) and nasal cavity volume (cm^3^) using acoustic rhinometry [20]. No statistical difference was reported in mean values for the sum of the left and right (L+R) first minimal cross-sectional area (cm^2^) and the second minimal cross-sectional area measurements (cm^2^) at any time point (start compared with five minutes, five minutes compared with 10 minutes, and start compared with 10 minutes). The mean values for the sum of nasal cavity volume (cm^3^) revealed no statistical differences, for any measurement parameter, between the start compared with five minutes (7.53 versus 7.26, MD 0.27); start minutes compared with 10 minutes (7.53 versus 6.88, MD 0.65); or five minutes compared with 10 minutes (7.26 versus 6.88, MD 0.38).

An observational study by Yamagiwa et al. enrolling 10 healthy adults compared five minutes of cooling of both feet using ice-water immersion at 0-4°C with the cooling of one hand and forearm (up to 23 cm proximal from middle fingertip) for the same duration, on the outcome of nasal cavity, mean volume, in mL, as measured by acoustic rhinometry [25]. Nasal cavity volume was measured before cooling, during cooling, and up to 40-60 minutes after five minutes of cooling. During the cooling phase, the foot-cooling group's nasal airway volume was significantly higher than before cooling in four of 10 (40%) subjects. In the hand-cooling arm (nine subjects), the nasal cavity volume increased significantly during cooling in only one of nine (11%) subjects. It was noted that measuring the average nasal cavity volume during each of the three periods did not provide an estimate of a rapid change in nasal volume during cooling. When volume changes were analyzed over time, most subjects were noted to increase their nasal volume just after the start of cooling, but this volume decreased before the end of the cooling period.

Additional Embase full-text record review

Seven additional full-text papers; five narratives [12,14,15,26,27], one editorial [28], and one technique were reviewed as they specifically addressed cryotherapy for epistaxis in the first aid setting (Table 3) [29]. All papers recommend direct pressure to the nostril between the thumb and index finger for five to 30 minutes. Six papers recommend applying cryotherapy to the face or nose (ice packs) or mouth (sucking); however, no evidence for these recommendations was provided [12,14,15,27-29]. Bird suggests that ice application to the nose does little to reduce blood flow; however, the author referenced this suggestion in a textbook citation [26].

**Table 3:** Additional Embase full-text paper characteristics and findings.

Author, year, country	Study design, setting/audience	Intervention concepts	Cryotherapy statements	Direct pressure application time
Bird, 1999, United Kingdom [26]	Narrative: A&E department	Direct pressure; Cryotherapy	“Local vasoconstriction induced by the application of ice to the nose, neck or mouth appears to do little in reducing blood flow …” p. 11	NA
Honeysett, 1982, United States [12]	Narrative: Nursing	Direct pressure; Patient position & reassurance; Cryotherapy	“… application of ice packs or cubes is useful as they can be soothing as well as causing local vasoconstriction with encourages the bleeding to stop.” p. 578	5 min
Ludman, 1981, United Kingdom [14]	Narrative: Clinical practice	Direct pressure; Cryotherapy	“… pinching the nostril between a finger and thumb and applying ice packs to the bridge of the nose.” p. 968	N/A
McLarnon and Carrie, 2015, United Kingdom [15]	Narrative: Clinical practice	Direct pressure; Patient position; Cryotherapy	“… alongside cooling with an ice pack on the nose or sucking an ice lolly if available.” p. 589	5-10 min Persistent, 20 min
Nicolas and Jassar, 2013, United Kingdom [28]	Editorial: Clinical practice	Direct pressure; Patient position; Cryotherapy	“… cold compress to the nose, is often used in addition to, sucking ice cubes, which is thought to help vasoconstrict the blood vessels within the nasal mucosa; however, there is little clinical evidence to support this as a treatment option.” p. 704	10 min
Shellenbarger, 2000, United States [27]	Narrative: Nursing	Direct pressure; Patient position; Cryotherapy	“… ice compresses over the middle face to promote vasoconstriction.” p. 50	5-30 min
Vaghela, 2005, United Kingdom [29]	Technique: A&E department	Direct pressure; Patient position; Cryotherapy	“… ice pack applied to the nasal bridge.” p. 261	20 min

Gray literature and ILCOR Resuscitation Sub-Council Guidelines review

Several Pubmed.gov and gray literature records were reviewed including two professional clinical guidelines [5,30], three endorsement statements [31-33], one systematic review [13], two narrative reviews [16,34], one practice guideline [35], one review [4], one protocol [17], and one website [36].

The American Academy of Otolaryngology-Head and Neck Surgery (AAO-HNS) recently recommended the use of nasal compression with "firm sustained compression to the lower third of the nose with or without the assistance of the patient or caregiver, for five minutes or longer" [5]. The AAO-HNS did not advocate for or address the utilization of cryotherapy as a first-line treatment. This statement is further endorsed by the American Academy of Physicians in 2019 [32], the American Academy of Pediatrics in 2020 [31], and the Society of Interventional Radiology [33]. Additionally, the Google.com gray literature search (i.e., "practice guideline" AND Epistaxis) identified 26/50 (52%) websites referencing back to the AAO-HNS clinical practice guideline for the management of epistaxis without recommending cryotherapy.

In 2017, The French Society of Otorhinolaryngology (SFORL) published guidelines for the first aid treatment of epistaxis in adults [30]. Similar to the AAO-HNS recommendations, the SFORL first aid guidelines for epistaxis include nasal cavity cleaning, the head raised slightly forward, and anterior bi-digital compression for 10 minutes, based on expert opinion. The SFORL guidelines also did not advocate for or address the utilization of cryotherapy as a first-aid treatment.

Also, in 2017, a systematic review by Khan et al. [13] of the initial assessment in the management of adults with epistaxis included reports on two randomized controlled trials on the effects of topical ice pack previously described in this scoping review [21,24]. Khan et al. concluded the application of an intra-oral ice pack is a simple first-aid measure with the potential to decrease bleeding severity and should be considered from the onset of epistaxis to the point of hospital care [13]. In contrast, evidence supporting the efficacy of the application of other topical ice packs was insufficient to make a recommendation.

Six gray literature records examined the use of cryotherapy as first aid treatment for epistaxis; specifically, the application of cryotherapy to the face or nose [16,17], sucking on ice [16,34], the application around [16] and to the back of the neck [12], or the forehead (Table 4) [34,35]. No evidence for these recommendations was provided in three records [16,17,36]. A narrative review by Wong and Anat suggested that ice packs around the neck and intra-oral ice significantly reduced nasal mucosa blood flow and could slow down nasal bleeding [34]. However, they referenced Porter et al. who measured blood flow in healthy adults [21]. Two reviews by Beck et al. and Record suggested the use of cryotherapy was inconclusive and controversial, citing work by Scheibe et al. and Teymoortash et al. [4,23,24,35].

**Table 4:** Gray literature study characteristics and findings.

Author, year, country	Study design, setting/audience	Intervention concepts	Cryotherapy statements	Direct pressure application time
Pope and Hobbs, 2005, United Kingdom [16]	Narrative: Clinical practice	Direct pressure; Patient position; Cryotherapy	“... improved by a cold compress or the patient sucking on ice.” p. 310	NA
Wong and Anat, 2018, Australia [34]	Narrative: Family practice	Direct pressure; Patient position; Cryotherapy	“Applying ice packs around the neck and having the patient suck on ice significantly reduces nasal mucosa blood flow and can slow down the bleeding.^7^” p. E16	10 min
Record, 2015, United States [35]	Practice guideline: Nursing	Direct pressure; Patient position; Cryotherapy	“Ice compresses to the forehead or neck may be used, but studies are inconclusive as to the usefulness of this maneuver (Teymoortash 2003; Scheibe, 2006).” p. 487	10 min
Upile et al., 2008, United Kingdom [17]	Protocol: United Kingdom Healthcare System, first aid	Direct pressure; Cryotherapy	“… pinching the whole of the cartilaginous tip of the nose for 30 min followed by another 30 min of pressure and pack of ice on bridge of nose if bleeding continued.” p. 1351	30 min + 30 min
Epistaxis, 2020, N/A [36]	Informational: Website	Direct pressure; Cryotherapy	“… putting an ice pack on to their forehead.” p. N/A	20 min
Beck, 2018, German [4]	Review: Primary and secondary care	Direct pressure; Patient position; Cryotherapy	“Local application of ice, e.g., at the back of the neck, is intended to encourage vasoconstriction of the blood vessels of the nose.” Its therapeutic value is a matter of debate and has been challenged in the literature (38).” p. 17	15-20 min

A search of the websites for the eight constituent resuscitation councils (and sub-councils) of ILCOR identified 29 first aid guideline statements with two sub-council guideline statements specifically addressing epistaxis. The 2000 American Heart Association first aid guidelines recommended pinching the nasal alae with the thumb and index finger to control bleeding; however, cryotherapy as an intervention is not addressed [19]. The 2017 Australian and New Zealand Committee on Resuscitation (ANZCOR) Guideline 9.1.1 states, pressure must be applied equally to both sides of the nose, over the soft part below the bony bridge (usually between the thumb and index finger) [37]. No mention of cryotherapy is made. Eleven guideline statement documents did not address epistaxis, three were inaccessible, seven offered no identifiable statement, and four could not be translated and offered no English documentation.

Discussion

This scoping review attempted to identify the current state of the literature regarding the use of cryotherapy as an intervention for the management of spontaneous epistaxis in the first aid setting. Given that most individuals with nasal bleeding experience a minor bleeding episode and opt for conservative first aid at home, it would be helpful to provide a recommendation for or against the use of a cryotherapy intervention for spontaneous nontraumatic epistaxis. However, we believe the usefulness of cryotherapy applied alone or in conjunction with compression to control active bleeding for spontaneous nontraumatic epistaxis remains unclear. 

Our scoping review found that most manuscripts and gray literature advocating cryotherapy for the management of spontaneous nontraumatic epistaxis in the first aid setting use expert opinion or indirect evidence derived from studies examining nasal submucosal temperature, nasal blood flow, and nasal blood volume in healthy subjects. The potential effect of cryotherapy is proposed to be the result of vasoconstriction within the nasal mucosa with the subsequent reduction of nasal blood flow and nasal blood volume.

The supporting studies were indirect and conducted using healthy adults (pooled sample n=44, 20-40 years, mean age 31 years reported in two of three studies) without epistaxis [21-23]. The studies included a limited number of participants and did not address the effect of cryotherapy on nasal blood flow and nasal blood volume in children or elderly adults or those with co-morbidities such as uncontrolled hypertension or concurrent anticoagulant use.

In reviewing the limitations of the three manuscripts, we noted that the methods of application of cryotherapy were inconsistent and that they were never applied directly to the nose, but instead to the forehead, in the mouth, around the neck, or in a combination of these techniques. We also identified that the cryotherapy application times varied between the three studies (three minutes, 10 minutes, 15 minutes) [21-23]. We did not identify any prospective, randomized trials comparing the efficacy of cryotherapy as an intervention for spontaneous nontraumatic epistaxis in the first aid setting.

This scoping review failed to identify studies critically evaluating the first aid management of epistaxis in adults and children. The lack of studies highlights research and knowledge gaps. There is a lack of clinical trials examining the effectiveness of cryotherapy (alone or in conjunction with nose pinching) in individuals with spontaneous or even traumatic epistaxis, in the first-aid setting. In addition, there are knowledge gaps regarding the effect of the addition of cryotherapy to nasal compression in persons with co-morbidities such as hypertension or anticoagulant use.

## Conclusions

This scoping review, conducted as part of the ILCOR continuous evidence evaluation process, found no direct evidence evaluating cryotherapy as an adjunctive intervention for spontaneous nontraumatic epistaxis in the first aid setting. Published manuscripts and gray literature records offer recommendations for the use of cryotherapy based on expert opinion or indirect evidence extrapolated from studies using healthy subjects. The lack of direct evidence found by this scoping review does not support the development of a systematic review. However, this scoping review does highlight the need for future research to better understand the role of cryotherapy as a first-aid strategy for spontaneous nontraumatic epistaxis.

## Appendices

Appendix 1 

**Table 5:** EMBASE.com (Embase + Medline) initial search query and results.

Approach	No.	Search string query (July 13, 2020)	Results
Nosebleed; nose near bleed (title or abstract) + first aid (title, abstract, keyword or index terms) + cold (title or abstract)	#1	“epistaxis”/de OR epistaxis:ti,ab OR nosebleed*:ti,ab OR ({[nose OR nasal OR rhino*] NEAR/2 [blood OR bleed$ OR bleeding OR haemorrhag* OR hemorrhag*]}:ti,ab)	25,792
	#2	“first aid”/de OR “emergency treatment”/de OR “emergency medicine”/de OR “first aid”:ti,ab,kw,de OR “first response”:ti,ab,kw,de OR “first responder$”:ti,ab,kw,de OR bystander$:ti,ab,kw,de OR “by stander*”:ti,ab,kw,de OR “wilderness medicine”:ti,ab,kw,de	89,878
	#3	ice:ti,ab OR cryo*:ti,ab OR cold:ti,ab OR cool:ti,ab OR cooling:ti,ab	337,941
	#4	#1 AND (#2 OR #3)	605
Nosebleed; nose (title) + cold (title)	#5	“epistaxis”/de OR epistaxis:ti OR nosebleed*:ti OR nose:ti OR nasal:ti OR rhino*:ti	106,480
	#6	ice:ti OR cryo*:ti OR cold:ti OR cool:ti OR cooling:ti	104,696
	#7	#5 AND #6	481
Nosebleed (title) + study design (trial; study)	#8	epistaxis:ti OR nosebleed$:ti OR “nose bleed$”:ti	3121
	#9	#8 AND (“clinical protocol”/de OR “clinical study”/de OR “clinical trial”/de OR “comparative effectiveness”/de OR “comparative study”/de OR “controlled clinical trial”/de OR “controlled study”/de OR “multicenter study”/de OR “randomized controlled trial”/de OR “randomized controlled trial topic”/de)	478
	#10	#4 OR #7 OR #9	1439
	#11	#10 NOT (rhinosinusitis:ti,ab,kw,de OR rhinorrhea:ti,ab,kw,de OR “common cold”:ti,ab,kw,de OR cryoablation:ti,ab,kw,de OR decongestant:ti,ab,kw,de OR rhinoplasty:ti,ab,kw,de OR ablation:ti,ab,kw,de OR tranexamic:ti,ab,kw,de OR ligation:ti,ab,kw,de)	1032
	#12	#11 NOT ({conference abstract}/lim OR {conference review}/lim OR {editorial}/lim OR {erratum}/lim OR {note}/lim OR {book}/lim OR “case report”/de)	739
	#13	#12 NOT ({“animal”/exp OR “nonhuman”/exp OR “rodent”/exp OR “animal experiment”/exp OR “experimental animal”/exp OR rat:ti,ab OR rats:ti,ab OR mouse:ti,ab OR mice:ti,ab OR dog$:ti,ab OR pig$:ti,ab OR porcine:ti,ab OR swine:ti,ab OR chick$:ti,ab} NOT “human”/exp)	669

Appendix 2 

**Table 6:** Cochrane library initial search query and results. MeSH: medical subject headings

No.	Search string query (July 13, 2020)	Results
1	MeSH descriptor: [Epistaxis] explode all trees	204
2	epistaxis:ti,ab OR nosebleed*:ti,ab OR ({nose:ti OR nasal:ti OR rhino*:ti} and {blood:ti or bleed*:ti or haemorrhage*:ti or hemorrhage*:ti})	8987
3	#1 or #2	9024
4	ice:ti,ab OR cryo*:ti,ab OR cold:ti,ab OR cool:ti,ab OR cooling:ti,ab	17,670
5	#3 and #4	207
6	epistaxis:ti	232
7	#5 or #6	439
8	#7 NOT (rhinosinusitis:ti,ab OR rhinorrhea:ti,ab OR “common cold”:ti,ab OR cryoablation:ti,ab OR decongestant:ti,ab OR rhinoplasty:ti,ab OR ablation:ti,ab)	309
	Cochrane reviews	2
	Cochrane protocols	1
	Trials	306

Appendix 3 

**Table 7:** EMBASE.com (Embase + Medline) follow-up search query and results.

No.	Search string query (January 14, 2021)	Results
1	“epistaxis”/de OR epistaxis:ti,ab OR nosebleed*:ti,ab OR ({[nose OR nasal OR rhino*] NEAR/2 [blood OR bleed$ OR bleeding OR haemorrhag* OR hemorrhag*]}:ti,ab)	26,730
2	“first aid”/de OR “emergency treatment”/de OR “emergency medicine”/de OR “first aid”:ti,ab,kw,de OR “first response”:ti,ab,kw,de OR “first responder$”:ti,ab,kw,de OR bystander$:ti,ab,kw,de OR “by stander*”:ti,ab,kw,de OR “wilderness medicine”:ti,ab,kw,de	92,792
3	ice:ti,ab OR cryo*:ti,ab OR cold:ti,ab OR cool:ti,ab OR cooling:ti,ab	350,738
4	#1 AND (#2 OR #3)	621
5	“epistaxis”/de OR epistaxis:ti OR nosebleed*:ti OR nose:ti OR nasal:ti OR rhino*:ti	109,911
6	ice:ti OR cryo*:ti OR cold:ti OR cool:ti OR cooling:ti	108,359
7	#5 AND #6	490
8	epistaxis:ti OR nosebleed$:ti OR “nose bleed$”:ti	3187
9	#8 AND (“clinical protocol”/de OR “clinical study”/de OR “clinical trial”/de OR “comparative effectiveness”/de OR “comparative study”/de OR “controlled clinical trial”/de OR “controlled study”/de OR “multicenter study”/de OR “randomized controlled trial”/de OR “randomized controlled trial topic”/de)	500
10	#4 OR #7 OR #9	1484
11	#10 NOT (rhinosinusitis:ti,ab,kw,de OR rhinorrhea:ti,ab,kw,de OR “common cold”:ti,ab,kw,de OR cryoablation:ti,ab,kw,de OR decongestant:ti,ab,kw,de OR rhinoplasty:ti,ab,kw,de OR ablation:ti,ab,kw,de OR tranexamic:ti,ab,kw,de OR ligation:ti,ab,kw,de)	1064
12	#11 NOT ({conference abstract}/lim OR {conference review}/lim OR {editorial}/lim OR {erratum}/lim OR {note}/lim OR {book}/lim OR “case report”/de)	759
13	#12 NOT ({“animal”/exp OR “nonhuman”/exp OR “rodent”/exp OR “animal experiment”/exp OR “experimental animal”/exp OR rat:ti,ab OR rats:ti,ab OR mouse:ti,ab OR mice:ti,ab OR dog$:ti,ab OR pig$:ti,ab OR porcine:ti,ab OR swine:ti,ab OR chick$:ti,ab} NOT “human”/exp)	688
14	#13 AND [12-7-2020]/sd	7

Appendix 4 

**Table 8:** Cochrane Library follow-up search query and results. *In July 2020, the string found 8987 records. Believe fewer trials were due to changes made to the CENTRAL database.

No.	Search string query (January 14, 2021)	Results
1	[mh epistaxis]	207
2	epistaxis:ti,ab OR nosebleed*:ti,ab OR ({nose:ti OR nasal:ti OR rhino*:ti} and {bloodti or bleed*:ti or haemorrhage*:ti or hemorrhage*:ti})	945
3	#1 or #2	1009
4	ice:ti,ab OR cryo*:ti,ab OR cold:ti,ab OR cool:ti,ab OR cooling:ti,ab	18699
5	#3 and #4	14
6	epistaxis:ti	244
7	#5 or #6	258
8	#7 NOT (rhinosinusitis:ti,ab OR rhinorrhea:ti,ab OR common cold:ti,ab OR cryoablation:ti,ab OR decongestant:ti,ab OR rhinoplasty:ti,ab OR ablation:ti,ab)	250
9	#8 with Publication Year from 2020 to 2021, with Cochrane Library publication date Between Jul 2020 and Jan 2021, in Trials	7
10	#8 with Cochrane library publication date between July 2020 and January 2021, in Cochrane reviews, Cochrane protocols	0

Appendix 5 

**Table 9:** PubMed clinical practice guideline and position statement search query and results. MeSH: medical subject headings

Approach	No.	Search string query (December 19, 2020)	Results
Title, abstract, keyword, or index terms	1	("guideline"{Publication Type} OR "guidelines as topic"{MeSH Terms} OR "guideline"{All Fields}) AND ("epistaxis"{MeSH Terms} OR "epistaxis"{All Fields})	58
	2	("protocol"{All Fields} OR "protocol s"{All Fields} OR "protocolized"{All Fields} OR "protocols"{All Fields}) AND ("epistaxis"{MeSH Terms} OR "epistaxis"{All Fields}) responder$:ti,ab,kw,de OR bystander$:ti,ab,kw,de OR “by stander*”:ti,ab,kw,de OR “wilderness medicine”:ti,ab,kw,de	211
			269

Appendix 6 

**Table 10:** Google.com literature search query and results.

Approach	No.	Search string query (December 21, 2020)	Results
Limited to past year, verbatim, first 50 hits	1	“practice guideline” AND Epistaxis	59,500
			59,500

Appendix 7 

**Table 11:** Google Scholar literature search query and results.

Approach	No.	Search string query (December 28, 2020)	Results
Limited to verbatim, first 100 hits	1	"First aid" AND Epistaxis AND ICE NOT Chronic NOT Posterior NOT Cautery NOT intranasal NOT Idiopathic NOT Packing NOT Silver NOT Tranexamic”);	3
	2	“First Aid Guidelines”	853
	3	"First aid" AND Nosebleed AND ICE OR Cryotherapy	930
			1786

Appendix 8 

**Table 12:** Resuscitation councils and sub-councils search query and results.

Approach	No.	Search string query (December 28, 2020)	Results
Hand search	1	American Heart Association	6
	2	European Resuscitation Council and associated Councils	10
	3	Heart and Stroke Foundation	3
	4	Australia and New Zealand Committee on Resuscitation (ANZCOR)	1
	5	Resuscitation Council of Asia and associated Councils	7
	6	Resuscitation Council of Southern Africa and associated Councils	1
	7	InterAmerican Heart Foundation	1
			29

## References

[1] Viehweg TL, Roberson JB, Hudson JW (2006). Epistaxis: diagnosis and treatment. J Oral Maxillofac Surg.

[2] McGarry GW, Moulton C (1993). The first aid management of epistaxis by accident and emergency department staff. Arch Emerg Med.

[3] Tabassom A, Cho JJ (2021). Epistaxis. https://www.ncbi.nlm.nih.gov/books/NBK435997/.

[4] Beck R, Sorge M, Schneider A, Dietz A (2018). Current approaches to epistaxis treatment in primary and secondary care. Dtsch Arztebl Int.

[5] Tunkel DE, Anne S, Payne SC (2020). Clinical practice guideline: nosebleed (epistaxis) executive summary. Otolaryngol Head Neck Surg.

[6] Kucik CJ, Clenney T (2005). Management of epistaxis. Am Fam Physician.

[7] Gifford TO, Orlandi RR (2008). Epistaxis. Otolaryngol Clin North Am.

[8] (2021). Clinical knowledge summaries: epistaxis (nosebleed) summary. https://cks.nice.org.uk/topics/epistaxis-nosebleeds/.

[9] (2021). Nosebleed. https://www.nhsinform.scot/illnesses-and-conditions/ears-nose-and-throat/nosebleed.

[10] American Heart Association (2005). Part 10: first aid. Circulation.

[11] Bishow RM (2003). Current approaches to the management of epistaxis. JAAPA.

[12] Honeysett J (1982). Epistaxis. Nurs Times.

[13] Khan M, Conroy K, Ubayasiri K (2017). Initial assessment in the management of adult epistaxis: systematic review. J Laryngol Otol.

[14] Ludman H (1981). ABC of ENT. Nose bleeds. Br Med J (Clin Res Ed).

[15] McLarnon CM, Carrie S (2015). Epistaxis. Surgery (Oxford).

[16] Pope LE, Hobbs CG (2005). Epistaxis: an update on current management. Postgrad Med J.

[17] Upile T, Jerjes W, Sipaul F, Maaytah ME, Singh S, Hopper C, Wright A (2008). A change in UK epistaxis management. Eur Arch Otorhinolaryngol.

[18] Drettner B (1964). Vascular reactions on the intake of food and drink of various temperatures. Acta Otolaryngol Suppl.

[19] American Heart Association (2000). Part 5: new guidelines for first aid. Circulation.

[20] Ozturk M, Mutlu F, Kara A, Derin S, Topdag M (2014). Evaluation of the effect of nasal dorsal skin cooling on nasal mucosa by acoustic rhinometry. J Laryngol Otol.

[21] Porter M, Marais J, Tolley N (1991). The effect of ice packs upon nasal mucosal blood flow. Acta Otolaryngol.

[22] Porter MJ (1991). A comparison between the effect of ice packs on the forehead and ice cubes in the mouth on nasal submucosal temperature. Rhinology.

[23] Scheibe M, Wüstenberg EG, Hüttenbrink KB, Zahnert T, Hummel T (2006). Studies on the effects of ice collars on nasal blood volume using optical rhinometry. Am J Rhinol.

[24] Teymoortash A, Sesterhenn A, Kress R, Sapundzhiev N, Werner JA (2003). Efficacy of ice packs in the management of epistaxis. Clin Otolaryngol Allied Sci.

[25] Yamagiwa M, Hilberg O, Pedersen OF, Lundqvist GR (1990). Evaluation of the effect of localized skin cooling on nasal airway volume by acoustic rhinometry. Am Rev Respir Dis.

[26] Bird D (1999). Managing epistaxis in A&E. Emerg Nurse.

[27] Shellenbarger T (2000). Nosebleeds: not just kids' stuff. RN.

[28] Nichols A, Jassar P (2013). Paediatric epistaxis: diagnosis and management. Int J Clin Pract.

[29] Vaghela HM (2005). Using a swimmer's nose clip in the treatment of epistaxis in the A&E department. Accid Emerg Nurs.

[30] Bequignon E, Vérillaud B, Robard L, Michel J, Prulière Escabasse V, Crampette L, Malard O (2017). Guidelines of the French Society of Otorhinolaryngology (SFORL). First-line treatment of epistaxis in adults. Eur Ann Otorhinolaryngol Head Neck Dis.

[31] American Academy of Pediatrics (2020). Clinical practice guideline: nosebleed (epistaxis). Pediatrics.

[32] (2021). Clinical practice guideline: nosebleed (epistaxis). https://www.acep.org/patient-care/clinical-policies/related-clinical-policy-resources/clinical-policies-from-outside-organizations-and-endorsed-by-acep/.

[33] (2021). Clinical practice guideline: nosebleed (epistaxis). https://www.sirweb.org/practice-resources/clinical-practice/guidelines-and-statements/neuro-and-spine/collaboration_endorsements/cpg_nosebleed-epistaxis/.

[34] Wong AS, Anat DS (2018). Epistaxis: a guide to assessment and management. J Fam Pract.

[35] Record S (2015). Practice guideline: epistaxis in children. J Pediatr Health Care.

[36] (2021). Epistaxis. http://emed.ie/HE-ENT/ENT/Epistaxis.php.

[37] (2021). ANZCOR guideline 9.1.1 - principles for the control of bleeding for first aiders. https://www.revive2survive.com.au/wp-content/uploads/2016/09/anzcor-guideline-9-1-1-bleeding-jan-16.pdf.

